# Motor Planning Error: Toward Measuring Cognitive Frailty in Older Adults Using Wearables

**DOI:** 10.3390/s18030926

**Published:** 2018-03-20

**Authors:** He Zhou, Hyoki Lee, Jessica Lee, Michael Schwenk, Bijan Najafi

**Affiliations:** 1 Interdisciplinary Consortium on Advanced Motion Performance (iCAMP), Michael E. DeBakey Department of Surgery, Baylor College of Medicine, Houston, TX 77030, USA; he.zhou2@bcm.edu (H.Z.); leehyoki@gmail.com (H.L.); jcl1@bcm.edu (J.L.); 2 Network Aging Research, Heidelberg University, 69115 Heidelberg, Germany; schwenk@nar.uni-heidelberg.de

**Keywords:** cognitive frailty, wearable, cognitive–motor impairment, Alzheimer’s disease, motor planning error, instrumented trail-making task, ankle reaching task, dual task walking

## Abstract

Practical tools which can be quickly administered are needed for measuring subtle changes in cognitive–motor performance over time. Frailty together with cognitive impairment, or ‘cognitive frailty’, are shown to be strong and independent predictors of cognitive decline over time. We have developed an interactive instrumented trail-making task (iTMT) platform, which allows quantification of motor planning error (MPE) through a series of ankle reaching tasks. In this study, we examined the accuracy of MPE in identifying cognitive frailty in older adults. Thirty-two older adults (age = 77.3 ± 9.1 years, body-mass-index = 25.3 ± 4.7 kg/m^2^, female = 38%) were recruited. Using either the Mini-Mental State Examination or Montreal Cognitive Assessment (MoCA), 16 subjects were classified as cognitive-intact and 16 were classified as cognitive-impaired. In addition, 12 young-healthy subjects (age = 26.0 ± 5.2 years, body-mass-index = 25.3 ± 3.9 kg/m^2^, female = 33%) were recruited to establish a healthy benchmark. Subjects completed the iTMT, using an ankle-worn sensor, which transforms ankle motion into navigation of a computer cursor. The iTMT task included reaching five indexed target circles (including numbers 1-to-3 and letters A&B placed in random order) on the computer-screen by moving the ankle-joint while standing. The ankle-sensor quantifies MPE through analysis of the pattern of ankle velocity. MPE was defined as percentage of time deviation between subject’s maximum ankle velocity and the optimal maximum ankle velocity, which is halfway through the reaching pathway. Data from gait tests, including single task and dual task walking, were also collected to determine cognitive–motor performance. The average MPE in young-healthy, elderly cognitive-intact, and elderly cognitive-impaired groups was 11.1 ± 5.7%, 20.3 ± 9.6%, and 34.1 ± 4.2% (*p* < 0.001), respectively. Large effect sizes (Cohen’s *d* = 1.17–4.56) were observed for discriminating between groups using MPE. Significant correlations were observed between the MPE and MoCA score (*r* = −0.670, *p* < 0.001) as well as between the MPE and dual task stride velocity (*r* = −0.584, *p* < 0.001). This study demonstrated feasibility and efficacy of estimating MPE from a practical wearable platform with promising results in identifying cognitive–motor impairment and potential application in assessing cognitive frailty. The proposed platform could be also used as an alternative to dual task walking test, where gait assessment may not be practical. Future studies need to confirm these observations in larger samples.

## 1. Introduction

Recent demographic changes have led to emergence of the so-called “dementia epidemic” [1]. Dementia causes great stress to medical, social, and informal care and is currently affecting approximately 47.5 million persons worldwide [2,3,4]. This number is projected to increase to 75.6 million by 2030 and 135.5 million by 2050 [3]. This has created an urgent need for a robust and quickly-administered cognitive assessment tool capable of identifying individuals in the earliest stages of cognitive decline and measuring subtle changes in cognitive–motor performance over time [5]. Early diagnosis of mild cognitive impairment (MCI) and detection (or ‘measuring’) of its subtle progression over time may allow early intervention and prevention of further cognitive and functional decline [6]. In this context, frailty together with cognitive impairment (known as ‘cognitive frailty’ [7]) has been shown to be a strong and independent predictor of cognitive decline over time [1,8,9].

The concept of ‘frailty’ is used to identify homeostenotic older adults with low physiological reserves and vulnerability to illness and high risk of disability, institutionalization, and death [10,11]. A recent systematic review identified 67 frailty instruments total; of these, nine were highly-cited (≥200 citations) [12]. Among these, the Physical Frailty Phenotype [10] was the most used frailty instrument in the research literature, followed by the Deficit Accumulation Index [11]. The Physical Frailty Phenotype is based on five criteria (weight loss, exhaustion, low physical activity, weak grip strength, and slow walking speed) [10]. The Deficit Accumulation Index is based on a count of 70 accumulated deficits, including the presence and/or severity of diseases, ability in activities of daily living, and physical signs from clinical and neurological examinations [11]. While both assessments have shown to be accurate for identifying older adults with low resilience and high vulnerability independent of comorbidities and disability [13,14], they all neglect assessing cognitive performance, including working memory and executive function. The cognitive assessment could add complementary information (in addition to frailty) to determine healthy aging trajectory as well as post intervention health outcomes. For example, previous studies have suggested that cognitive performance is an independent risk factor influencing adverse health outcomes after major surgical intervention [15,16].

A recent study by Kelaiditi et al. [9] demonstrated that measures of frailty can be used to identify individuals with Alzheimer’s disease (AD) who have a greater risk of cognitive decline and loss of independence. An association between cognitive impairment and specific frailty criteria such as gait speed has been demonstrated [17]. Similarly, several studies have suggested that cognitive and motor declines are independent key predictors of dementia in the geriatric population [8,18,19].

Despite this evidence described above, cognitive impairment and frailty are still assessed and studied separately, mainly because conventional cognitive performance assessment tools do not take into account motor performance (an indicator of frailty), and vice versa. In addition, the current motor or cognitive performance assessment tools often neglect characterization of anticipatory motor planning, an important factor that influences ability to accomplish instrumental activities of daily living (iADL) [20,21,22].

Studies based on motor learning paradigms highlight the importance of characterizing anticipatory motor planning as an important factor that influences daily motor skills and cognitive performance [23,24]. Most of iADLs rely on the individual’s anticipatory planning abilities to appropriately plan movements prior to executing them. For example, to successfully accomplish a behavioral goal such as reaching for a piece of fruit, two problems must be solved: to select which fruit to reach for and to specify the parameters of the reach, such as its direction and extent [24]. Studies on primary motor cortex, premotor cortex, and supplementary motor area have demonstrated that human brains can plan and control physical movement ahead of physical body action [25,26,27]. It is well established that anticipatory planning abilities improve as a function of age, reaching adult-like levels in late childhood to early adolescence. It then rapidly declines to levels observed in young children, in very old adults, particularly in those with frailty and/or cognitive impairment [28]. It has been postulated that the decrease in anticipatory motor planning abilities in older adults is associated with decline in cognitive skills [28], a theory supported by brain imaging research in which an association between deterioration in frontal and parietal white matter and fine motor dexterity was reported [29,30]. Recently, Stockel and colleagues [28] have demonstrated that up to 64% of the variance in motor planning performance across age groups can be explained by the cognitive functions of processing speed, response planning, and cognitive flexibility. They have postulated that anticipatory motor planning abilities are strongly influenced by cognitive control processes, which seem to be key mechanisms compensating for age related decline.

To address the practical barriers for clinical use of cognitive–motor assessment tools as well as incorporating a practical method to quantify motor planning, we have developed a novel cognitive frailty measurement tool call instrumented trail-making task (iTMT). The iTMT is based on the combination of a low-cost wearable sensor attached to patient’s lower shin and interactive interface technology. The design of the system is inspired by the conventional trail-making test (TMT), which is a useful test to determine cognitive impairment [31]. The second component of the system is an ankle reaching test performed in a virtual environment, which allows for quantification of motor performance and motor planning error. The proposed system provides a unique opportunity to determine cognitive frailty by enabling simultaneous and rapid measurement of both cognitive and motor performance. In our previous study [32], we have demonstrated the effectiveness of the iTMT to determine cognitive impairment by measuring the time duration needed to complete the test. In addition, we have demonstrated that by measuring the ankle velocity during the reaching task, we can also distinguish pre-frail individuals and frail individuals from non-frail individuals. In the present study, we extended our iTMT approach by quantifying anticipatory motor planning error. We extracted the motor planning error (MPE) through measuring ankle velocity patterns during the ankle reaching task. Our hypothesis was that; due to anticipatory motor planning performance decline associated with cognitive impairment, older adults with cognitive impairment would have iTMT MPEs larger than those of elderly without cognitive impairment as well as those of young-healthy subjects.

## 2. Methods

### 2.1. Study Population

Thirty-two ambulatory older adults (age 65 years or older) were recruited in this study. According to the Mini-Mental State Examination (MMSE) (cutoff score of 23 or less [33]) or Montreal Cognitive Assessment (MoCA) (cutoff score of 25 or less [34]), 16 subjects were classified as cognitive-intact, and 16 were classified as cognitive-impaired. To compare the iTMT results between young and older adults, as well as to establish a healthy benchmark, 12 young-healthy ambulatory subjects with ages ranging from 21 to 34 years were also recruited. Subjects were excluded from the study if they were unable to walk a distance of 20 m with or without walking assistance; had visual problem, limiting their ability to interact with a computer screen; had lower extremity problems, limiting their ability to perform ankle movement needed for the purpose of this study; or had severe balance impairment, limiting their ability to independently stand. Those who could stand behind a chair and perform the iTMT test by holding the chair were not excluded. All subjects signed a consent form for this study. This study was approved by the local institutional review boards.

### 2.2. Demographic Information

All subjects’ demographics were collected, including age, gender, body mass, height, body-mass-index (BMI), history of falls, and history of depression. Subjects self-reported any fall incidents and number of falls in the past year. The Center for Epidemiologic Studies Depression (CES-D) short-version scale was used to measure self-reported depression symptoms. A cutoff of CES-D score of 16 or greater was used to identify subjects with depression.

### 2.3. Instrumented Trail-Making Task (iTMT)

**Figure 1:**
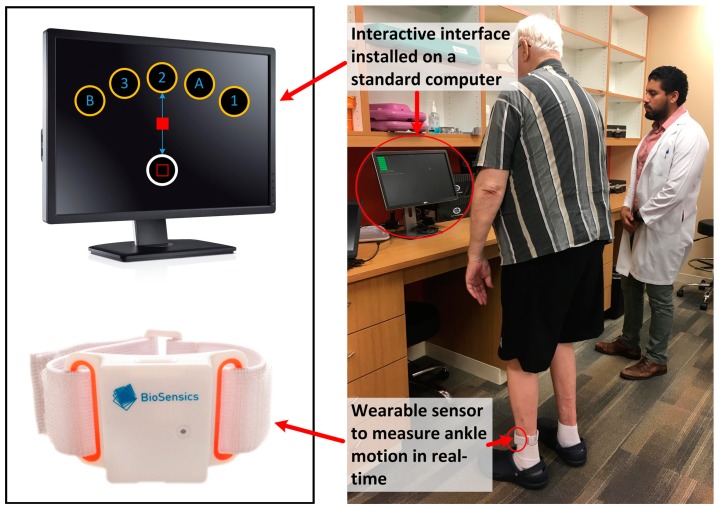
An illustration of the instrumented trail-making task (iTMT) platform with both numbers (1, 2, and 3) and letters (A and B) randomized in the target circles. One inertial sensor—including a triaxial accelerometer, a triaxial gyroscope, and a triaxial magnetometer—was attached to the subject’s lower shin using a comfortable elastic band. The sensor allows measurement of three-dimensional motion of the ankle joint in real time. The instantaneous measured joint angle with a sample frequency of 100-Hz was wirelessly transferred to a computer, using low-power Bluetooth, to create an interactive interface for the purpose of the interactive iTMT test. For safety purposes, a research coordinator was in the room supervising the iTMT test at all times. After starting the iTMT test, the research coordinator did not provide any guidance. The interactive interface provided the necessary guidance and instruction to complete the test.

We designed the iTMT platform (Figure 1) based on a wearable sensor (LEGSys™, Biosensics LLC, Watertown, MA, USA), which has a triaxial accelerometer, triaxial gyroscope, and triaxial magnetometer for the purpose of estimating angles and positions. The wearable sensor was attached to the lower shin for tracking three dimensional ankle motion (Figure 1). The real-time data was acquired and transmitted at 100-Hz frequency to an interactive computer interface (developed based on MATLAB^®^ 2013b and Psychophysics Toolbox Version 2.54) installed on a standard computer (Figure 1). The subject could navigate a cursor from a home circle to target circles appearing on the computer screen using ankle rotation.

In the iTMT test, six circles appeared on the screen: one home circle in white and five target circles in yellow. The target circles were located in a fanwise position in front of the home circle (Figure 1). The five target circles were randomly marked with numbers (1, 2, and 3) and letters (A and B). At the beginning of the iTMT test, the cursor position was automatically calibrated to the center of the home circle. The iTMT required weight shifting in forward/backward or sideward/diagonal direction, while standing with both feet on the ground in front of the computer screen (Figure 1). To perform the iTMT, the subject would control the movement of ankle joints while maintaining balance. This could be achieved by moving the hip joint in opposite direction of ankle motion (reciprocal compensatory strategy) [35]. Ankle motions on the left foot and right foot are consistent, therefore by attaching just one sensor on one lower shin, three dimensional ankle motion can be tracked. By rotating the ankle joint, the subject could navigate the cursor to the center of target circles with alternating numbers and letters. Specifically, the subject navigated the cursor to the center of the first target circle (with the number ‘1’ inside). The target circle would explode with a reward sound once the cursor stopped in the middle of the target circle. Then the subject navigated the cursor back to the center of the home circle, then to the second target circle (with the letter ‘A’), then back to the home circle, then to the third target circle (with the number ‘2’), and so forth until the end. To efficiently accomplish the test with minimum time to reach all targets, the subject should have optimum motor planning prior to executing the task. For example, to successfully reach a target, two problems should be solved: to select which target to reach for (i.e., working memory task) and to specify the parameters of the reach, such as its direction and extent. In addition, to minimize the time to reach a target, the subject should determine optimum time to decelerate ankle movement so as stop perfectly at the center of the target without re-adjustment.

### 2.4. iTMT Motor Planning Error

**Figure 2:**
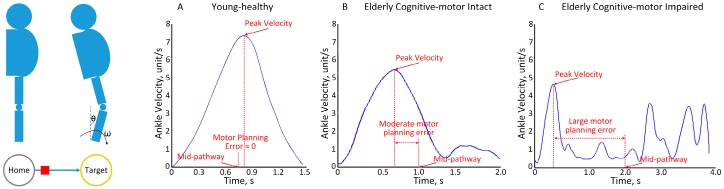
An illustration of the iTMT MPE. (**A**) Ankle velocity curve of a typical young-healthy subject during the ankle reaching, (**B**) ankle velocity curve of a typical elderly cognitive–motor intact subject during the ankle reaching, (**C**) ankle velocity curve of a typical elderly cognitive–motor impaired subject during the ankle reaching.

We extracted the motor planning error based on ankle velocity pattern measurements during the ankle reaching task. MPE was defined as percentage of time deviation between subject’s maximum ankle velocity and the optimal maximum ankle velocity, which is halfway through the reaching pathway (Figure 2). In summary, to minimize the iTMT time, which requires bringing a computer cursor (square shape) from a home circle and stopping at the middle of the target circle (which was defined as the end of a reaching task), the subject should reach maximum ankle velocity at the middle of the reaching pathway and then decelerate to be able to stop at the middle of the target without the need for re-adjustment. Therefore, the ideal ankle velocity curve is a bell shape (indicator of a healthy feedforward performance). Based on our definition, the iTMT MPE can range from 0 to 50%. 0% indicates that the peak ankle velocity appears at the mid-pathway (optimum motor planning). 50% indicates that the peak ankle velocity appears at the beginning or end of target reaching. Figure 2A–C show ankle velocity curves of a typical young-healthy subject, elderly cognitive–motor intact subject, and elderly cognitive–motor impaired subject, respectively. For the typical young-healthy subject with good cognitive perception coordinated with good motor performance, the peak ankle velocity appears very close to the mid-pathway, and the iTMT MPE is very small (Figure 2A). For the typical elderly cognitive–motor intact subject, the peak ankle velocity is lower than the young-healthy subject, and the iTMT MPE is moderate (Figure 2B). For the typical elderly cognitive–motor impaired subject with poor cognition and uncoordinated motion, the peak ankle velocity appears very early or very late, and the iTMT MPE is large. Multiple ankle velocity peaks can be observed in Figure 2C.

### 2.5. iTMT Time

The method for measuring the iTMT time has been described in detail in [32]. In summary, at the beginning of the iTMT test, when the cursor position is automatically calibrated to the center of the home circle, the computer program starts recording the time. At the end of the iTMT test, when the cursor position stops at the center of the last target circle (with the number ‘3’), the computer program stops recording the time. The recorded time period is defined as the iTMT time, which represents the total time a subject uses to complete reaching to all five indexed target circles in the correct order. Our previous study suggests that the iTMT time result is correlated with MoCA test result (*r* = −0.598, *p* = 0.001) and is effective at discriminating between those with and without cognitive impairment with large effect size (Cohen’s *d* effect size = 1.02, *p* = 0.015) [32]. We have also demonstrated that the iTMT time represents mainly cognitive performance and corresponds poorly with motor performance (e.g., gait speed) [32]. To compare this marker with the innovative iTMT MPE proposed in this study, iTMT time was collected for all subjects.

### 2.6. Walking Test

For all subjects, wearable sensors were attached to both the left and right lower legs (LegSys™, BioSensics, Watertown, MA, USA) to measure single task and dual task gait performances. Subjects were asked to walk with their habitual gait speed for 20 m with no cognitive task (single task walking). Then, they were asked to repeat the test while loudly counting backward from a random number (dual task walking: walking + working memory test) [2,36]. Gait speed was calculated using validated algorithms [37,38,39].

### 2.7. Statistical Analysis

Age, height, body mass, BMI, single task walking stride velocity, and dual task walking stride velocity for each group were described using both mean ± standard deviation. Gender, history of falls, and history of depression were expressed as count (percentage). The Shapiro–Wilk test was applied to test normality of the data. One-way analysis of variance was employed for pairwise comparison according to the scale of the investigated variable and the distribution of the data. Analysis of covariance was used to compare the iTMT MPE and the iTMT time, with and without adjustment for age and BMI, among groups. Fisher’s least significant difference-based post hoc test was performed for pairwise comparison to explore significant main effects and interactions. Cohen’s *d* effect size was calculated to assess the magnitude of difference between each group. Values ranging from 0.20 to 0.49 indicate small, and values between 0.50 and 0.79 indicate medium. Values ranging from 0.80 to 1.29 indicate large effects, and values above 1.30 indicate very large effects. Values less than 0.20 are considered as having no noticeable effect. Spearman’s correlation coefficients were used to evaluate the degree of association between the iTMT MPEs and conventional dual task walking stride velocity, used as a reference. *p* < 0.050 was considered statistically significant. All statistical analyses were performed using IBM SPSS Statistics 24 (IBM, Chicago, IL, USA).

#### Sample Size Calculation

We estimated prior sample size and power by performing a post hoc analysis based on the observed effect size in the previous iTMT study [32]. Considering the observed effect size of 1.02 for the iTMT time difference between cognitive-intact and cognitive-impaired in [32], to achieve a minimum power of 80%, a minimum sample size of 13 subjects per group are required to observe a statistical significant of 5% or lower using two tails independent sample comparisons.

## 3. Results

### 3.1. Study Population

Table 1 summarizes demographic and clinical data. The elderly subjects’ ages ranged from 60 to 93 years, while the young-healthy subjects’ ages ranged from 21 to 34 years. No difference between the elderly cognitive-intact group and elderly cognitive-impaired group was observed for demographic information, including gender, age, height, body mass, and BMI (*p* > 0.050). For the clinical examinations including history of fall and depression, no between-group difference was observed (*p* > 0.050). Motor performance based on single task walking did not show significant difference between the elderly cognitive-intact group and elderly cognitive-impaired group. The only significant difference between the two groups was the dual task walking stride velocity (*p* = 0.031). Compared to the elderly cognitive-intact group, the young-healthy group had significantly increased single task and dual task walking stride velocities (*p* < 0.001 and *p* = 0.001).

### 3.2. iTMT Motor Planning Error and iTMT Time among Groups

**Figure 3:**
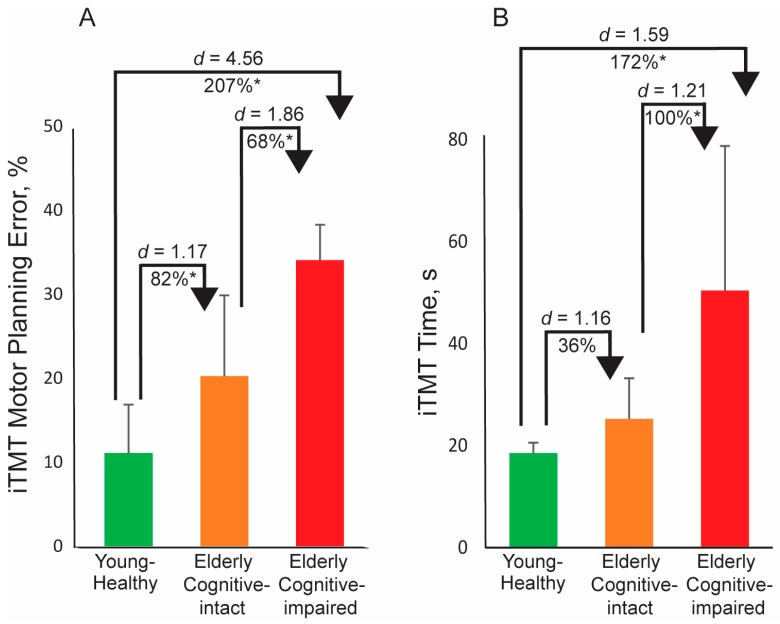
The iTMT derived parameters for all groups, including young-healthy group, elderly cognitive-intact group, and elderly cognitive-impaired group. (**A**) The iTMT MPE comparison; (**B**) the iTMT time comparison. Error bar represents the standard error. ‘*’ denotes when the pairwise group comparison achieved a statistically significant level (*p* < 0.050). *d* denotes Cohen’s *d* effect size.

Table 2 summarizes the iTMT MPE and the iTMT time for different groups, including young-healthy subjects, elderly cognitive-intact subjects, as well as elderly cognitive-impaired subjects. Table 3 and Figure 3 summarize between-group comparisons with and without adjustment for age and BMI. The iTMT MPE generated during the test was 20.3 ± 9.6% in the elderly cognitive-intact group and was significantly increased on average by 68% in the elderly cognitive-impaired group (*d* = 1.86, *p* < 0.001, Figure 3A). The iTMT time spent by the elderly cognitive-intact group to complete the test was 25.2 ± 7.9s. It was significantly increased on average by 100% in the elderly cognitive-impaired group (*d* = 1.21, *p* < 0.001, Figure 3B). When compared with the elderly groups, a much lower iTMT MPE (11.1 ± 5.7%, Figure 3A) and a much shorter iTMT time (18.5 ± 2.1s, Figure 3B) was observed for the young-healthy group. When compared with the iTMT time, the iTMT MPE had much larger effect sizes to discriminate between the three groups (Table 3). After adjusting the results for age and BMI, the between-group difference achieved statistical significance only when comparing between the elderly cognitive-intact and elderly cognitive-impaired groups for both iTMT MPE (*d* = 1.26, *p* < 0.001) and iTMT time (*d* = 0.98, *p* < 0.001) (Table 3).

### 3.3. Association between iTMT Motor Planning Errors and Conventional Cognitive Assessment

**Figure 4:**
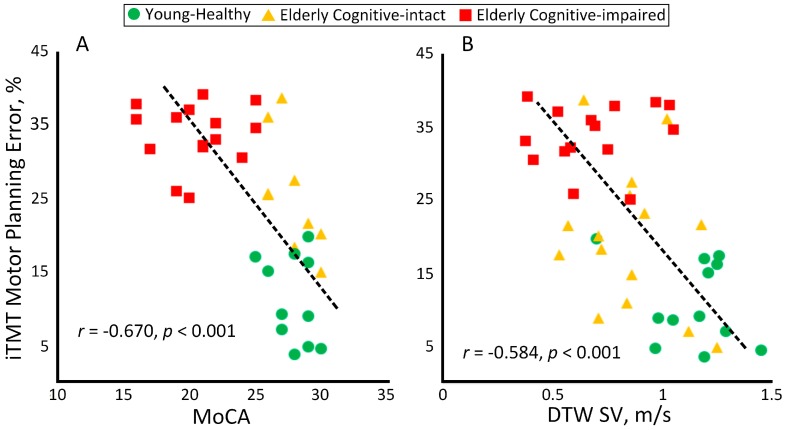
Correlation between the iTMT MPE and (**A**) MoCA test score and (**B**) dual task walking stride velocity (DTW SV).

Figure 4 illustrates the correlation between the iTMT platform and conventional cognitive assessments. In summary, a high correlation was observed between the iTMT MPE and MoCA score (*r* = −0.670, *p* < 0.001, Figure 4A). On the same note, a high correlation was also observed between the iTMT MPE and dual task walking stride velocity (*r* = −0.584, *p* < 0.001, Figure 4B), indicating that the iTMT MPE may be used as a surrogate for the dual task walking test.

## 4. Discussion

Cognitive and motor impairments are independent key predictors of further decline in cognitive performance, life independency, and Alzheimer’s disease among geriatric population [8,18,19]. In this study, we investigated the feasibility and efficacy of estimating motor planning error from a practical wearable platform with promising results in identifying cognitive–motor impairment in older adults. We were able to confirm our hypothesis that the iTMT MPE is able to discriminate between elderly cognitive-intact and elderly cognitive-impaired groups. The iTMT MPE value is derived from the ankle velocity signals during trail-making and ankle-reaching tasks for examination of both cognitive and motor abilities. In other words, the ankle velocity pattern can provide information on both cognition and motor ability of patients. For example, the velocity pattern of the elderly cognitive-impaired group fluctuated more than that of the elderly cognitive-intact group (Figure 2). This may be due to the fact that both trail-making and ankle-reaching tasks require performing automatic corrections under voluntary control by the brain in order to sharpen target localization [40]. Therefore, the noisy velocity pattern signifies the decrease of motor planning skills in geriatric populations with cognitive impairment.

Cognitive impairment is a symptom that makes it difficult for patients to remember, learn new things, concentrate, or make decisions. While cognitive impairment can be identified with pencil- and paper-based screening tools, such as the MoCA, MMSE, and conventional TMT, these assessments are semi-subjective, time consuming, and insensitive to subtle changes in cognitive frailty. Their accuracy is highly dependent on the examiner’s experience and the patient’s education level [32,41]. Computerized versions of conventional cognitive screening tools have improved the utility of such measurements. However, they are not capable of monitoring motor performance (an essential component of frailty) and anticipatory planning abilities (an essential component for successfully accomplishment of instrumental activities of daily living) thus are not able to detect cognitive frailty, a known predictor of speed of cognitive decline over time. The dual task paradigm can be used to screen for both cognitive and motor performance and identify both cognitive impairment and frailty [2,36]. The most stablished dual task paradigm is dual task walking. Dual task walking can isolate the cognitive control component of locomotion and expose cognitive deficits through the evaluation of activities that simultaneously demand attentional resources. In addition, dual task walking test is sensitive in determining frailty [36]. However, many older adults have mobility impairment and high risk of falling, making the administration of walking test challenging, in particular in busy clinical settings. In addition, limits on space and time in busy clinics can influence the execution of such tests [42]. Even if gait is assessed over short walking distance to accommodate space limitation, it is debated whether dual-task walking test is reliable over a short walking distance (less than 20 m) [38,43]. The proposed ankle reaching task derived MPE addresses the aforementioned limitations associated with pencil- and paper-based screening tools as well as dual task gait. The iTMT platform consists of a low-cost wearable sensor combined with an interactive interface installable on any standard computer [32]. It is simple, short, safe, and easy to manage by non-expert users [32]. The iTMT platform does not require walking test. It allows long-term quantification of anticipatory motor planning regardless of setting.

In an early study [32], we demonstrated that the iTMT time is an efficient marker to discriminate among age-matched healthy, amnestic mild cognitive impairment, and Alzheimer’s disease groups. However, the iTMT time is a global marker of the trail-making performance. It is the total time that a subject needs to complete the iTMT test and can be influenced by many factors such as the time a subject needs to search and locate targets, the speed a subject can rotate the ankle, the number of mistakes a subject makes during the test, etc. Therefore, the iTMT time is not a specific maker of cognitive–motor impairment. It may not be as sensitive as the iTMT MPE in identifying cognitive–motor impairment and assessing cognitive frailty. The iTMT MPE is derived from ankle velocity measurements during the trail-making test. It is a specific marker which can evaluate both cognitive and motor abilities equally and simultaneously. We collected both iTMT markers in the present study and compared them in Table 2 and Table 3. Although Table 2 shows that both iTMT markers possessed significant differences between the three groups (*p* < 0.001), Table 3 shows that the iTMT MPE had a larger effect size than the iTMT time for discriminating among the three groups, mainly because of relatively lower intra group variation, in particular among the elderly group with cognitive impairment. For instance, when examining the difference between older adults with and without cognitive impairment, the effect size was increased from 1.21 (large effect size) by using the iTMT time to 1.86 (very large effect size) by using the iTMT MPE. In addition, only the iTMT MPE can discriminate between young-healthy and elderly cognitive-intact without adjustment for age. This may be because the major difference between these two groups exists in the motor domain caused by aging. Thus, we may speculate that the iTMT MPE is a cognitive–motor sensitive metric, and may be able to determine motor deficit between groups with comparable cognitive functions. Before adjusting for age and BMI, the iTMT MPE was able to discriminate between all three groups. After adjusting for age and BMI, the difference between young-healthy and older adults was diminished irrespective of cognitive status. However, when comparing between older adults with and without cognitive impairment, the between-group difference remained significant after adjustment. This may suggest that the iTMT MPE is sensitive to age but also able to determine cognitive impairment in the same age categories.

One of the unique opportunities of the iTMT platform is its ability to determine frailty status. In another study, we demonstrated that iTMT parameters were also able to identify presence and absence of frailty phenotypes (Cohen’s *d* effect size = 0.81–1.56). Specifically, the iTMT maximum ankle reaching velocity was associated with slowness, changes in ankle velocity from the first reaching task to the last reaching task was an indicator of exhaustion, ankle reaching moment was a representation of weakness, and jerkiness of ankle movement was associated with inactivity. These parameters allow identification of non-frail, pre-frail, and frail older adults, as identified by the Physical Frailty Phenotype [10], with large to very large effect sizes (*d* = 1.04–3.14). Together with results of the current study in which we demonstrated that motor planning error is an indicator of cognitive impairment, it is postulated that the iTMT platform is able to determine cognitive frailty, a strong predictor of elderly dependency in the near future [8]. Recent studies suggest that frailty is also a significant predictor of cognitive decline over a short period of time. For example, Kelaiditi et al. [9] demonstrated that frail patients with AD have almost twice the decline in cognitive performance over one year follow up than AD patients without frailty. The same study concluded that a one-unit (0.033 points) increase in the frailty index (indicator of cumulative deficits) corresponds to significant and clinically relevant cognitive decline, after adjusting for age, sex, and years of education (0.63–4.63 points on the MMSE, *p* = 0.010).

Another advantage of the iTMT platform is that it uses wearable technology, instead of camera or force platform, to evaluate motor-cognitive performance in older adults. Unlike low-cost camera-based motion tracking system (e.g., Microsoft Kinect), wearable sensors do not require a continuous unobstructed sightline; and thus the test could be administrated behind of a chair to provide support if needed. In addition, the examiner could be next to the subject to provide safety, unlike with the camera-based system. This safety feature is especially important during the iTMT in older adults, in particular, those with cognitive impairment and dementia, who have increased fall risk. The force platform is similar to wearable sensor, which is not limited by continuous unobstructed sightline. However it has other limitations making it unsuitable for motor planning error assessment. For instance, commercially available and low-cost force platforms (e.g., Nintendo Wii Fit) restrict the base of support during testing, which is unsuitable for obese patients or those who need large base of support to maintain balance during dynamic tasks [44]. Furthermore, wearable sensors enable measuring kinematics of body joint of interest and thus diversify the tests. Force platforms, however, are limited to measure only center of pressure or ground reaction force without any information from joint angles.

### Limitations and Future Directions

Despite the advantages of the iTMT platform enabling us to prescreen for cognitive–motor impairment and cognitive frailty, there are some limitations in this study. Our subjects were recruited from specialized outpatient clinics (e.g., cancer clinic, vascular clinic, dementia clinic, etc.) instead of older adults dwelling communities. Thus our sample may not represent general elderly population. The instruments (MMSE or MoCA) used to identify cognitive impairment in different clinics were not identical. Future study should be done on different days and be administered by different examiners in order to assess test–retest reliability. In addition, this study analyzed the iTMT MPE while standing, which disqualifies bedbound and non-ambulatory patients. This study invites further analysis of the motor planning error while sitting or lying down in order to identify cognitive–motor impairment in bedbound patients.

## 5. Conclusions

In conclusion, we have demonstrated that motor planning error, which could be estimated from a low-cost and simple wearable platform, is able to simultaneously determine cognitive and motor impairments. Based on our observations, the test is feasible and practical even for those with severe cognitive impairment (MoCA < 17) and poor motor performance (single task walking stride velocity < 0.5m/s). The test is fast (taking less than one minute on average, excluding preparation and sensor attachment), it requires a single inertial sensor (i.e., gyroscope) and low-cost standard computer or tablet, and is reliable as demonstrated in our previous study [32]. Despite the simplicity, it can provide important clinical information about cognitive–motor impairment and cognitive frailty in geriatric populations. The proposed iTMT platform could also be used as an alternative test for dual task gait, which is often impractical in busy clinics and homes, where the space for gait assessment is not available, and for patients with limited mobility (e.g., need to walk with walking accessories). Thus, it can easily be used for both community dwelling elderly as well as those in busy hospital settings.

## Figures and Tables

**Table 1:** General characteristics of the study population.

	Elderly Cognitive-Intact (N)	Elderly Cognitive-Impaired (P)	*p*-Value (P vs. N)	Young-Healthy (H)	*p*-Value (N vs. H)
Number of subject, n	16	16	-	12	-
Female, n (%)	7.0 (44)	5.0 (31)	0.481	4.0 (33)	0.593
Age, years	75.6 ± 9.5	79.0 ± 8.6	0.292	26.0 ± 5.2	**<0.001**
Height, cm	170.0 ± 9.8	168.5 ± 12.8	0.723	170.9 ± 9.4	0.798
Body mass, kg	69.9 ± 14.6	77.7 ± 20.9	0.240	74.3 ± 15.3	0.450
BMI, kg/m^2^	24.0 ± 3.4	26.7 ± 5.5	0.113	25.3 ± 3.9	0.387
History of fall, n (%)	4.0 (25)	7.0 (44)	0.279	-	-
Depression, n (%)	4.0 (25)	2.0 (13)	0.381	-	-
STW SV, m/s	1.00 ± 0.18	0.87 ± 0.21	0.075	1.27 ± 0.15	**<0.001**
DTW SV, m/s	0.85 ± 0.21	0.68 ± 0.22	**0.031**	1.14 ± 0.21	**0.001**

BMI: Body-Mass-Index; STW: Single Task Walking; DTW: Dual Task Walking; SV: Stride Velocity. Depression was assessed by Center for Epidemiologic Studies Depression (CES-D) score with a cutoff of 16 or greater. Significant difference between groups were indicated in bold.

**Table 2:** iTMT derived parameters for different groups.

	Young-Healthy	Elderly Cognitive-Intact	Elderly Cognitive-Impaired	*p*-Value
iTMT Motor Planning Error, %	11.1 ± 5.7	20.3 ± 9.6	34.1 ± 4.2	**<0.001**
iTMT Time, s	18.5 ± 2.1	25.2 ± 7.9	50.4 ± 28.3	**<0.001**

iTMT: instrumented trail-making task. Significant difference between groups were indicated in bold.

**Table 3:** Between-group comparison of iTMT motor planning error and iTMT time with and without adjustment for age and BMI

	iTMT Motor Planning Error						iTMT Time					
	Without Adjustment			With Adjustment for Age and BMI			Without Adjustment			With Adjustment for Age and BMI		
	Difference Mean (%)	*p*-Value	*d*	Difference Mean (%)	*p*-Value	*d*	Difference Mean (%)	*p*-Value	*d*	Difference Mean (%)	*p*-Value	*d*
Elderly cognitive-intact vs. young-healthy	9.2 (82)	**0.001**	1.17	−4.3 (20)	0.547	0.29	6.7 (36)	0.331	1.16	−2.8 (11)	0.886	0.07
Elderly cognitive-impaired vs. young-healthy	22.9 (207)	**<0.001**	4.56	8.6 (41)	0.260	0.57	31.9 (172)	**<0.001**	1.59	24.2 (98)	0.238	0.61
Elderly cognitive-impaired vs. elderly cognitive-intact	13.8 (68)	**<0.001**	1.86	12.9 (77)	**<0.001**	1.26	25.2 (100)	**<0.001**	1.21	27.0 (122)	**<0.001**	0.98

iTMT: instrumented trail-making test. Significant difference between groups were indicated in bold. Effect sizes were calculated as Cohen’s *d.*

## References

[1] Kelaiditi E., Cesari M., Canevelli M., van Kan G.A., Ousset P.J., Gillette-Guyonnet S., Ritz P., Duveau F., Soto M.E., Provencher V. (2013). Cognitive frailty: Rational and definition from an (I.A.N.A./I.A.G.G.) international consensus group. J. Nutr. Health Aging.

[2] Bahureksa L., Najafi B., Saleh A., Sabbagh M., Coon D., Mohler M.J., Schwenk M. (2017). The Impact of Mild Cognitive Impairment on Gait and Balance: A Systematic Review and Meta-Analysis of Studies Using Instrumented Assessment. Gerontology.

[3] World Health Organization (WHO) (2016). Mental Health and Older Adults.

[4] Cornelis E., Gorus E., Beyer I., Bautmans I., De Vriendt P. (2017). Early diagnosis of mild cognitive impairment and mild dementia through basic and instrumental activities of daily living: Development of a new evaluation tool. PLoS Med..

[5] Brooks L.G., Loewenstein D.A. (2010). Assessing the progression of mild cognitive impairment to Alzheimer’s disease: Current trends and future directions. Alzheimers Res. Ther..

[6] Dierckx E., Engelborghs S., De Raedt R., De Deyn P.P., Ponjaert-Kristoffersen I. (2007). Mild cognitive impairment: What’s in a name?. Gerontology.

[7] Shimada H., Makizako H., Tsutsumimoto K., Doi T., Lee S., Suzuki T. (2018). Cognitive Frailty and Incidence of Dementia in Older Persons. J. Prev. Alzheimers Dis..

[8] Ruan Q., Yu Z., Chen M., Bao Z., Li J., He W. (2015). Cognitive frailty, a novel target for the prevention of elderly dependency. Ageing Res. Rev..

[9] Kelaiditi E., Canevelli M., Andrieu S., Del Campo N., Soto M.E., Vellas B., Cesari M. (2016). Impact of Cholinergic Treatment Use Study/DSA Group. Frailty Index and Cognitive Decline in Alzheimer’s Disease: Data from the Impact of Cholinergic Treatment USe Study. J. Am. Geriatr. Soc..

[10] Fried L.P., Tangen C.M., Walston J., Newman A.B., Hirsch C., Gottdiener J., Seeman T., Tracy R., Kop W.J., Burke G. (2001). Frailty in older adults: Evidence for a phenotype. J. Gerontol. Ser. A Biol. Sci. Med. Sci..

[11] Rockwood K., Andrew M., Mitnitski A. (2007). A comparison of two approaches to measuring frailty in elderly people. J. Gerontol. Ser. A Biol. Sci. Med. Sci..

[12] Buta B.J., Walston J.D., Godino J.G., Park M., Kalyani R.R., Xue Q.L., Bandeen-Roche K., Varadhan R. (2016). Frailty assessment instruments: Systematic characterization of the uses and contexts of highly-cited instruments. Ageing Res. Rev..

[13] Clegg A., Young J., Iliffe S., Rikkert M.O., Rockwood K. (2013). Frailty in elderly people. Lancet.

[14] Walston J. (2017). Frailty in older adults. Oxford Textbook of Geriatric Medicine.

[15] Montero M., Najafi B., Hinko V., Hoegliner S., Rahemi H., Enriquez A., Chung J., Barshes N., Gilani R., Mills J. (2017). Using Frailty and Cognitive Assessment to Predict Adverse Events After Major Vascular Intervention: Application of Wearable Technologies. J. Vasc. Surg..

[16] Millar K., Asbury A., Murray G. (2001). Pre-existing cognitive impairment as a factor influencing outcome after cardiac surgery. Br. J. Anaesth..

[17] Fougère B., Daumas M., Lilamand M., Sourdet S., Delrieu J., Vellas B., van Kan G.A. (2017). Association between frailty and cognitive impairment: Cross-sectional data from Toulouse frailty day hospital. J. Am. Med. Dir. Assoc..

[18] Verghese J., Robbins M., Holtzer R., Zimmerman M., Wang C., Xue X., Lipton R.B. (2008). Gait dysfunction in mild cognitive impairment syndromes. J. Am. Geriatr. Soc..

[19] Boyle P.A., Capurso C., D’Introno A., Colacicco A.M., Capurso A., Solfrizzi V. (2006). Mild cognitive impairment: risk of Alzheimer disease and rate of cognitive decline. Neurology.

[20] (2010). Patterns of loss of basic activities of daily living in Alzheimer patients: A cross-sectional study of the French REAL cohort. Dement. Geriatr. Cogn. Disord..

[21] Liang F.W., Chan W., Chen P.J., Zimmerman C., Waring S., Doody R. (2016). Cognitively-Related Basic Activities of Daily Living Impairment Greatly Increases the Risk of Death in Alzheimers Disease. PLoS ONE.

[22] Nguyen H., Lebel K., Bogard S., Goubault E., Boissy P., Clinicians Q.P.N., Duval C. (2017). Using Inertial Sensors to Automatically Detect and Segment Activities of Daily Living in People with Parkinson’s Disease. IEEE Trans. Neural Syst. Rehabil. Eng..

[23] Seidler R.D., Kwak Y., Fling B.W., Bernard J.A. (2013). Neurocognitive mechanisms of error-based motor learning. Adv. Exp. Med. Biol..

[24] Cisek P. (2006). Integrated neural processes for defining potential actions and deciding between them: A computational model. J. Neurosci..

[25] Tanji J., Shima K. (1994). Role for supplementary motor area cells in planning several movements ahead. Nature.

[26] Halsband U., Ito N., Tanji J., Freund H.-J. (1993). The role of premotor cortex and the supplementary motor area in the temporal control of movement in man. Brain.

[27] Shibasaki H., Sadato N., Lyshkow H., Yonekura Y., Honda M., Nagamine T., Suwazono S., Magata Y., Ikeda A., Miyazaki M. (1993). Both primary motor cortex and supplementary motor area play an important role in complex finger movement. Brain.

[28] Stockel T., Wunsch K., Hughes C.M.L. (2017). Age-Related Decline in Anticipatory Motor Planning and Its Relation to Cognitive and Motor Skill Proficiency. Front. Aging Neurosci..

[29] Raz N., Gunning-Dixon F., Head D., Rodrigue K.M., Williamson A., Acker J.D. (2004). Aging, sexual dimorphism, and hemispheric asymmetry of the cerebral cortex: Replicability of regional differences in volume. Neurobiol. Aging.

[30] Fazekas F., Ropele S., Enzinger C., Gorani F., Seewann A., Petrovic K., Schmidt R. (2005). MTI of white matter hyperintensities. Brain.

[31] Tombaugh T.N. (2004). Trail Making Test A and B: Normative data stratified by age and education. Arch. Clin. Neuropsychol..

[32] Zhou H., Sabbagh M., Wyman R., Liebsack C., Kunik M.E., Najafi B. (2017). Instrumented Trail-Making Task to Differentiate Persons with No Cognitive Impairment, Amnestic Mild Cognitive Impairment, and Alzheimer Disease: A Proof of Concept Study. Gerontology.

[33] Cockrell J.R., Folstein M.F. (2002). Mini-mental state examination. Principles and Practice of Geriatric Psychiatry.

[34] Nasreddine Z.S., Phillips N.A., Bedirian V., Charbonneau S., Whitehead V., Collin I., Cummings J.L., Chertkow H. (2005). The Montreal Cognitive Assessment, MoCA: A brief screening tool for mild cognitive impairment. J. Am. Geriatr. Soc..

[35] Najafi B., Horn D., Marclay S., Crews R.T., Wu S., Wrobel J.S. (2010). Assessing postural control and postural control strategy in diabetes patients using innovative and wearable technology. J. Diabetes Sci. Technol..

[36] Schwenk M., Howe C., Saleh A., Mohler J., Grewal G., Armstrong D., Najafi B. (2014). Frailty and technology: A systematic review of gait analysis in those with frailty. Gerontology.

[37] Grewal G., Sayeed R., Yeschek S., Menzies R.A., Talal T.K., Lavery L.A., Armstrong D.G., Najafi B. (2012). Virtualizing the assessment: A novel pragmatic paradigm to evaluate lower extremity joint perception in diabetes. Gerontology.

[38] Najafi B., Helbostad J.L., Moe-Nilssen R., Zijlstra W., Aminian K. (2009). Does walking strategy in older people change as a function of walking distance?. Gait Posture.

[39] Aminian K., Najafi B., Bula C., Leyvraz P.F., Robert P. (2002). Spatio-temporal parameters of gait measured by an ambulatory system using miniature gyroscopes. J. Biomech..

[40] Gaveau V., Pisella L., Priot A.E., Fukui T., Rossetti Y., Pélisson D., Prablanc C. (2014). Automatic online control of motor adjustments in reaching and grasping. Neuropsychologia.

[41] Lin F., Vance D.E., Gleason C.E., Heidrich S.M. (2012). Caring for older adults with mild cognitive impairment: an update for nurses. J. Gerontol. Nurs..

[42] Razjouyan J., Grewal G.S., Rishel C., Parthasarathy S., Mohler J., Najafi B. (2017). Activity monitoring and heart rate variability as indicators of fall risk: Proof-of-concept for application of wearable sensors in the acute care setting. J. Gerontol. Nurs..

[43] Lindemann U., Najafi B., Zijlstra W., Hauer K., Muche R., Becker C., Aminian K. (2008). Distance to achieve steady state walking speed in frail elderly persons. Gait Posture.

[44] Gerling K.M., Schild J., Masuch M. Exergaming for elderly: Analyzing player experience and performance. Proceedings of the Mensch & Computer.

